# Longitudinal association of breakfast and midnight snacks with depressive symptoms in China multi-ethnic adolescents

**DOI:** 10.3389/fpsyt.2025.1651630

**Published:** 2025-10-09

**Authors:** Honglv Xu, Gaohong Zhang, Zihan Liu, Xiaolu Xue, Dongyue Hu, Jieru Yang, Jing Jia, Xuemei Zhang

**Affiliations:** ^1^ School of Medicine, Kunming University, Kunming, China; ^2^ Community Nursing Research Team of Kunming University, Kunming, Yunnan, China; ^3^ School of Nursing, Yunnan University of Chinese Medicine, Kunming, China

**Keywords:** adolescent, depressive symptoms, breakfast, midnight snacks, longitudinal study

## Abstract

**Introduction:**

Studies have suggested a link between dietary behavior and adolescent depressive symptoms, but longitudinal data are scarce. This study examines the longitudinal association of breakfast and midnight snacks consumption with depressive symptoms among multi-ethnic adolescents in China.

**Methods:**

From October 2022 to October 2024, 1,693 middle school students (52.3% females) from Yunnan Province participated in five follow-up surveys (T1-T5) conducted every six months. Breakfast and midnight snacks consumption were assessed using questionnaires, and depressive symptoms were measured using Children’s Depression Inventory. The latent growth curve model was analyzed using Mplus software to assess the potential growth trajectories of breakfast days, midnight snacks days, and depressive symptom scores across five time points. The generalized estimation equation model was applied to examine the association between the number of breakfast and midnight snacks days and depressive symptom scores. Two models were established: Model 1 was unadjusted, without controlling any variables; Model 2 was adjusted for demographic variables and other potential confounders influencing depressive symptoms. A restricted cubic spline analysis was used to examine the relationship between the number of breakfast days per week, midnight snacks days per week, and depressive symptoms.

**Results:**

The prevalence of depressive symptoms increased from 26.3% at T1 to 37.3% at T5 (*P* < 0.01). After adjusting for confounders, breakfast frequency (*β* = -0.71, 95%*CI*: -0.87-0.56) and midnight snacks frequency in males (*β* = 0.39, 95%*CI*: 0.24 - 0.55) and females (*β* = -0.77, 95%*CI*: -0.92 - -0.63; *β* = 0.17, 95%*CI*: 0.02 - 0.32) were associated with depressive symptoms (all *P* < 0.05). Males eating breakfast and midnight snacks fewer than three days and more than four days, respectively, per week, and females eating breakfast and midnight snacks fewer than four days and more than two day, respectively, per week, had an increased risk of depressive symptoms.

**Discussion:**

Skipping breakfast and eating midnight snacks are related to depressive symptoms in multi-ethnic Chinese adolescents. Addressing unhealthy eating behaviors is critical for preventing and mitigating adolescent depressive symptoms.

## Introduction

Adolescent depression is a significant public health concern. This developmental stage is marked by rapid social, emotional, and cognitive changes, as well as major life transitions (1). Adolescence is also the peak period for depression onset (2) and represents a substantial disease burden in this age group (3, 4). Furthermore, depression is a key risk factor for self-harm and suicidal behaviors among adolescents (5, 6). Globally, the incidence and prevalence of depressive symptoms in adolescents are increasing (7–9). A representative study in Germany reported a prevalence of 18.4%, with both the severity and frequency of depressive symptoms being higher in females than in males. Additionally, depressive symptoms were found to increase with age (10). The prevalence of depressive symptoms among adolescents in the United exceeds 30% (11). Numerous studies have examined the prevalence of depressive symptoms among adolescents in developing countries. In Malaysia and India, reported prevalence rates range from 26.2% to 52.3%. In Bangladesh, 25.0% of adolescents experience depressive symptoms, with a higher prevalence in females (30.0%) compared to males (19.0%) (12). In China, the prevalence of depressive symptoms among adolescents is close to 40% (13). However, data on the prevalence of depressive symptoms among multi-ethnic adolescents in western China remain limited. Existing research suggests that depressive symptoms are influenced by multiple factors, including academic pressure, family stress, physical activity, screen time, and physical literacy (14–18). Recently, the relationship between dietary behaviors and depressive symptoms in adolescents has gained increasing attention.

Emerging evidence suggests that healthy dietary patterns, such as adherence to the Mediterranean diet, are negatively associated with depressive symptoms (19). In contrast, unhealthy dietary behaviors— including frequent snacking, skipping breakfast, consuming sugar-sweetened beverages, and eating ultra-processed foods —are positively linked to depressive symptoms in adolescents (20–23). Previous research has primarily examined the relationship between breakfast consumption and adolescent mental health (24, 25). Systematic reviews and meta-analyses indicate that skipping breakfast is positively associated with an increased risk of depressive symptoms in adolescents (25). In contrast, regular breakfast consumption (≥6 days per week) among Japanese adolescents is negatively associated with depressive symptoms (26, 27). Studies conducted in Spain, Turkey, and Bangladesh have identified skipping breakfast as a predictor of depressive symptoms. Adolescents who skip breakfast or consume it fewer than five days per week have a significantly higher incidence of depressive symptoms compared to those who eat breakfast regularly (12, 28, 29). However, few cross-sectional studies in China have examined the relationship between breakfast consumption and depressive symptoms in adolescents (30). Among Chinese adolescents aged 12–17 years, inconsistent breakfast consumption is a significant risk factor for mental health problems, with a more pronounced effect in females (31). Compared to those who consume breakfast ≥6 per week, the risk of depressive symptoms is higher in adolescents who eat breakfast 2–5 days per week (*OR* = 1.76) and those who do so ≤1 day per week (*OR* = 3.78) (32). Additionally, a study in eastern China among adolescents aged 11–19 years found that skipping breakfast (OR = 2.56) was independently associated with depressive symptoms (33).

Few studies have examined midnight snacks consumption among adolescents (34). Midnight snacks, also referred to as nighttime eating, typically involves consuming food between 9:00 PM and 04:00 AM, in addition to the three main daily meals. This behavior includes a variety of foods, such as snacks, vegetables, desserts, and other small meals. Previous research suggests that midnight snacks are common among adolescents, with reported prevalence rates of 9.0% in Canada (35), 16.6% in Bangladesh (36), and 29.7% in Palestine (37). Notably, the prevalence of midnight snacks among Chinese adolescents is significantly higher (72.5%) (38). Previous studies suggest associations between adolescent dietary disorders, intuitive eating, nighttime eating syndrome, and mental health (39–41). However, research on the relationship between midnight snacks and depressive symptoms in adolescents remains unlimited. In Palestinian adolescents, nighttime eating was linked to mental health issues, including depressive symptoms (*OR* = 4.18) (37). Additionally, a cross-sectional study of adolescents aged 12–13 years found that nighttime eating was associated with severe depressive symptoms (*OR* = 1.04) (35). A cross-sectional study in Turkey found that adolescents who frequently consumed midnight snacks reported higher levels of depressive symptoms (42). Similarly, studies in the United States and Switzerland indicated that depressive symptoms were more prevalent among adolescents diagnosed with night-eating syndrome (43, 44). Additionally, among Chinese children and adolescents in grades 1-9, those who ate before bedtime had a 1.28 times higher risk of depressive symptoms compared to those who did not (45).

While some studies have examined the relationship between breakfast and midnight snacks behaviors and adolescent depressive symptoms, research on multi-ethnic adolescents in China remains scarce. Yunnan Province, located in southwest China, is a major multi-ethnic region. Due to the influence of diverse food cultures and economic situations, adolescents in this area may be more likely to skip breakfast and consume midnight snacks. This study hypothesizes a longitudinal association between skipping breakfast, midnight snacks, and depressive symptoms among multi-ethnic adolescents in China. To test this hypothesis, we conducted a follow-up survey in Yunnan Province.

## Materials and methods

### Participants

**Figure 1:**
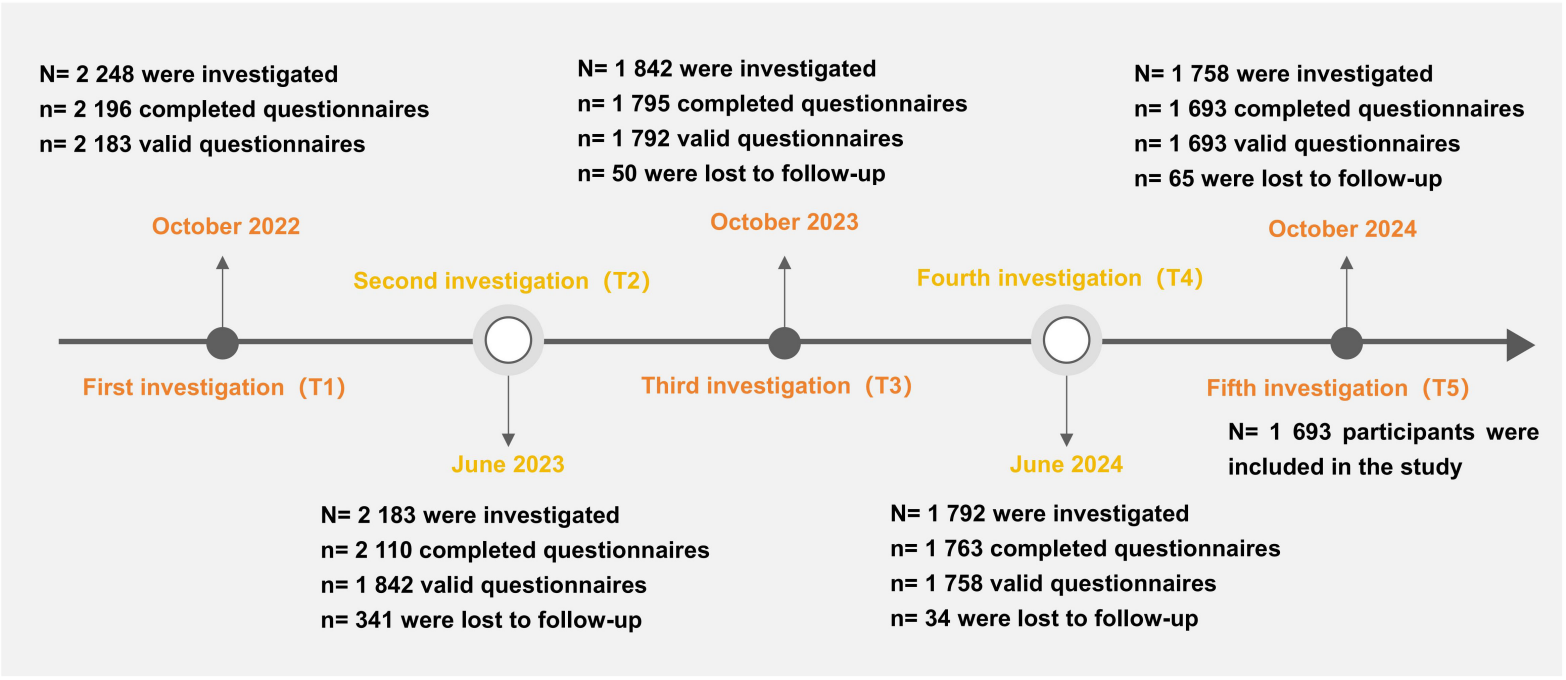
Flow chart of participants.

A longitudinal questionnaire survey was conducted among 1,693 middle school students from four schools in three regions of Yunnan Province, China. The baseline survey (T1) took place in October 2022, followed by an assessment every six months. The subsequent follow-up surveys were conducted in June 2023 (T2), October 2023 (T3), June 2024 (T4), and October 2024 (T5). The attrition rates for each follow-up were 15.6%, 2.7%, 1.7%, and 3.7%, respectively. Ultimately, 1,693 participants were included in the final analysis. A flowchart depicting the inclusion of study participants is presented in **Figure 1**. The average age the participants was (12.5 ± 0.5) years, with the minimum age being 12 years and the maximum 15 years. The final sample consisted of 808 males (47.7%), and 885 females (52.3%). The participants comprised 629 Han (37.2%), 628 Yi (37.1%), 66 Hani (3.9%), 143 Lahu (8.4%), 78 Dai (4.6%), and 149 individuals from other ethnic groups (8.8%). Among them, 1,278 (75.5%) resided in rural areas, while 415 (24.5%) were from urban areas. In terms of family structure, 1,391 participants (82.2%) came from two-parent families, 166 (9.8%) from single-parent families, 110 (6.5%) from blended families, and 26 (1.5%) from other family types. Additionally, 321 participants (19.0%) were only children. Regarding socioeconomic status, participants self-reported as follows: 49 (2.9%) as “worse,” 180 (10.6%) as “poor,” 1,132 (66.9%) as “medium,” 246 (14.5%) as “good,” and 86 (5.1%) as “better.” **Table 1** displays the distribution of demographic variables in relation to the prevalence of depressive symptoms. The study was in accordance with the requirement of the World Medical Association Declaration of Helsinki and granted with consent from the students, schools, and parents. The questionnaire survey was conducted anonymously, with voluntary participation from all teenage respondents. Participants retained the right to withdraw from the follow-up survey at any time. All collected data were treated with strict confidentiality and used exclusively for data analysis in this study, with no other applications. The study was approved by the Medical Ethics Committee of the School of Medicine, Kunming University (Approval Number: 20210222).

**Table 1:** The distribution of the positive rate of depressive symptoms in adolescents.

Variables	Category	T1				T2				T3				T4				T5				
		Depressive symptomS	Normal	*χ^2^*	*P*	Depressive symptomS	Normal	*χ^2^*	*P*	Depressive symptomS	Normal	*χ^2^*	*P*	Depressive symptomS		Normal	*χ^2^*	*P*	Depressive symptomS	Normal	*χ^2^*	*P*
Sex	Male	175	633	17.07	<0.01	203	605	27.47	<0.01	232	576	19.41	<0.01	260		548	9.66	0.002	255	553	21.57	<0.01
	Female	270	615			327	558			344	541			349		536			376	509		
Residence	Rural	357	921	7.32	0.007	417	861	4.25	0.039	449	829	2.87	0.091	465		813	0.39	0.534	479	799	0.10	0.755
	Urban	88	327			113	302			127	288			144		271			152	263		
Age(year)	12	243	716	1.24	0.539	311	648	1.34	0.511	328	631	0.07	0.965	357		602	1.55	0.460	374	585	2.92	0.232
	13	195	517			212	500			241	471			244		468			250	462		
	14-15	7	15			7	15			7	15			8		14			7	15		
Ethnicity	Han	145	484	44.77	<0.01	198	431	2.45	0.785	200	429	9.36	0.096	219		410	2.05	0.842	231	398	2.35	0.799
	Hani	28	38			26	40			32	34			25		41			29	37		
	Yi	142	486			191	437			217	411			226		402			230	398		
	Lahu	63	80			44	99			45	98			49		94			52	91		
	Dai	29	49			23	55			25	53			30		48			33	45		
	Other	38	111			48	101			57	92			60		89			56	93		
The number of close friends	0	15	18	77.40	<0.01	12	21	13.40	0.009	14	19	9.04	0.060	15		18	9.97	0.041	15	18	8.10	0.088
	1-2	89	85			74	100			75	99			69		105			78	96		
	3-4	84	209			95	198			101	192			115		178			117	176		
	5-6	60	248			94	214			102	206			122		186			108	200		
	≥7	197	688			255	630			284	601			288		597			313	572		
The only child in the family	Yes	80	241	0.38	0.538	97	224	0.22	0.641	106	215	0.18	0.674	113		208	0.10	0.750	115	206	0.35	0.552
	No	365	1007			433	939			470	902			496		876			516	856		
Family type	Two-parent family	351	1040	5.39	0.146	430	961	0.99	0.804	467	924	0.92	0.822	494		897	3.98	0.264	517	874	1.35	0.717
	One-parent family	48	118			55	111			61	105			59		107			64	102		
	Combined family	37	73			35	75			38	72			42		68			38	72		
	Other	9	17			10	16			10	16			14		12			12	14		
Self-perceived socioeconomic status	Worse	18	31	48.24	<0.01	19	30	20.93	<0.01	28	21	33.40	<0.01	23		26	6.83	0.145	27	22	17.20	0.002
	Poor	82	98			78	102			84	96			73		107			81	99		
	Medium	283	849			351	781			376	756			400		732			416	716		
	Good	47	199			64	182			67	179			89		157			85	161		
	Better	15	71			18	68			21	65			24		62			22	64		
Father's education level	Illiteracy	55	95	11.92	0.018	50	100	1.68	0.794	52	98	1.68	0.794	60		90	5.91	0.206	54	96	2.00	0.736
	Elementary school	116	299			135	280			148	267			153		262			160	255		
	Secondary school	196	593			237	552			268	521			276		513			292	497		
	High school	51	179			76	154			76	154			90		140			90	140		
	University	27	82			32	77			32	77			30		79			35	74		
Mother's education level	Illiteracy	63	131	7.04	0.134	61	133	2.10	0.717	73	121	3.16	0.531	73		121	2.14	0.711	73	121	2.17	0.704
	Elementary school	123	310			147	286			154	279			164		269			151	282		
	Secondary school	190	594			236	548			262	522			275		509			305	479		
	High school	43	131			52	122			52	122			63		111			62	112		
	University	26	82			34	74			35	73			34		74			40	68		
Father's occupation	Civil servant	237	628	4.41	0.491	262	603	10.39	0.065	305	560	5.30	0.381	300		565	14.43	0.013	322	543	7.98	0.157
	Worker	29	85			33	81			31	83			39		75			37	77		
	Staff	87	253			117	223			117	223			142		198			139	201		
	Merchant	29	119			38	110			45	103			39		109			43	105		
	Farmer	16	40			26	30			23	33			26		30			23	33		
	Other	47	123			54	116			55	115			63		107			67	103		
Mother's occupation	Civil servant	270	701	3.77	0.583	299	672	6.13	0.293	344	627	4.47	0.484	347		624	4.22	0.518	366	605	2.94	0.709
	Worker	25	83			27	81			31	77			32		76			37	71		
	Staff	42	137			62	117			62	117			68		111			68	111		
	Merchant	42	128			48	122			49	121			57		113			55	115		
	Farmer	15	57			24	48			23	49			28		44			28	44		
	Other	51	142			70	123			67	126			77		116			77	116		

### Methods

#### Survey methods

A cross-sectional questionnaire survey was conducted among middle school students across 11 counties in Yunnan Province using random cluster sampling. The details of this methodology have been described in our previously published work (46). Based on the cross-sectional survey, four middle schools with high multi-ethnic representation and strong participant compliance were selected for the longitudinal follow-up study. All seventh-grade (Grade 7) students from the selected schools participated in the survey. The adolescents completed a self-administered questionnaire in a classroom setting. The survey collected data on demographic characteristics (e.g., gender, age, ethnicity, only child status, family residence, self-rated family economic status, family structure, number of close friends, parental education levels, and parental occupations). Additionally, it assessed dietary behaviors (breakfast and midnight snacks), depressive symptoms, and potential confounding factors influencing adolescents’ depression, including family changes, hospitalization history, screen time, smoking, alcohol consumption, physical activity, family history of depression, self-rated academic pressure impact of the COVID-19 pandemic. Trained surveyors administered the survey in person, addressing participants’ questions face-to-face. Adolescents completed the questionnaire independently without discussing responses with peers. The process took approximately 15–20 minutes. Surveyors reviewed the completed questionnaires upon collection, promptly addressing any missing responses.

### Evaluation of depressive symptoms

Depressive symptoms were assessed using the Chinese version of the Children’s Depression Inventory (CDI), a widely used self-report scale for measuring depression in children and adolescents (47, 48). The CDI evaluates depressive mood and behavior in individuals aged 7–17 years, based on self-reported experiences over the past two weeks. The inventory consists of 27 items across five dimensions: anhedonia, negative mood, negative self-esteem, ineffectiveness, and interpersonal problems. Each item is rated on a 3-point scale (“occasionally,” “often” and “always”), with scores ranging from 0 to 2 per item, resulting in a total possible score of 0-54. Higher scores indicate more severe depressive symptoms. A total CDI score ≥19 was considered indicative of depressive symptoms, while a score < 19 was classified as negative. Studies on Chinese children and adolescents (aged 7–17 years) have demonstrated that the CDI has good reliability and validity, with a Cronbach’s α of 0.84 (49). Research on Chinese middle school students further supports the CDI’s high reliability and validity, confirming its suitability for assessing depressive symptoms in this population (Cronbach’s α coefficient of 0.853) (50). Cronbach’s α coefficients at T1 –T5 were 0.861, 0.892, 0.900, 0.893 and 0.904, respectively, indicating strong internal consistency.

### Breakfast behavior and midnight snacks behavior evaluation

Adolescent dietary behaviors were assessed using a dietary frequency questionnaire developed by the research group, which has been repeatedly validated for good reliability and validity (46, 51). Firstly, a pilot test was conducted in 131 middle school students before the field survey to assess participant understanding of the questionnaire. The pilot test results showed that middle school students were able to fully understand the content of the questionnaire, and the average time taken to complete the questionnaire was 16 minutes. Secondly, in each follow-up survey, we evaluated the reliability and validity of the dietary frequency questionnaire, the Cronbach’s α coefficients at T1 –T5 were 0.921, 0.936, 0.942, 0.945, and 0.940, respectively.

Midnight snacks behavior refers to the behavior of consumption of food between 21:00 and 04:00, in addition to the three main meals of the day. This includes various snacks, desserts, and other food items. Breakfast behavior refers to the consumption of the first meal of the day, typically between 06:00 and 08:00. This study the breakfast and midnight snacks behaviors of multi-ethnic adolescents. Participants were asked the following questions (1): “How many days did you eat breakfast in the past week?” Response options included (1) 0 days (2), 1–2 days (3), 3–4 days (4), 5–6 days (5), 7 days (2). “How many days did you consume midnight snacks (including snacks) before going to bed in the past week?” The response options included (1) 0 days (2), 1–2 days (3), 3–4 days (4), 5–6 days (5), 7 days. Adolescents selected the number of days they consumed breakfast and midnight snacks based on their actual eating habits.

### Statistical analysis

Data were entered into a database using EpiData 3.0. After data verification, statistical analyses were conducted using SPSS (version 23.0; SPSS Inc., Chicago, IL, USA), R (version 4.3.1), and Mplus (version 7.4). Descriptive statistics, *χ^2^* tests, and generalized estimation equation (GEE) models were performed in SPSS. Restricted cubic spline analysis was conducted using R software. The latent growth curve model (LGCM) was analyzed using Mplus software to assess the potential growth trajectories of breakfast days, midnight snacks days, and depressive symptom scores across five time points. Intercept and slope factors were included as observation indices. A *χ^2^* test was used to compare differences in the detection rates of depressive symptoms among college students with different demographic characteristics over time. The generalized estimation equation (GEE) model was applied to examine the association between the number of breakfast and midnight snacks days and depressive symptom scores. In the model, breakfast days and the number of midnight snacks per week were treated as independent variables, while depressive symptom scores were the dependent variable. Both independent and dependent variables were continuous variables. Two models were established: Model 1, which was unadjusted, without controlling any variables; Model 2, which was adjusted for demographic variables and other potential confounders influencing depressive symptoms, followed by an association analysis. The identification of confounding factors mainly stems from the variables reported in the literature that have evidence suggesting they may affect adolescent depression symptoms. The adjustment method is to include both the confounding variables and the analysis variables (the days of eating breakfast per week and the days of eating midnight snacks per week) in the model as independent variables. A restricted cubic spline analysis was used to examine the relationship between the number of breakfast days per week, midnight snacks days per week, and depressive symptoms. The statistical significance level was set at α = 0.05.

## Results

### The growth trajectories of breakfast and midnight snacks consumption and depressive symptoms among adolescents

**Figure 2:**
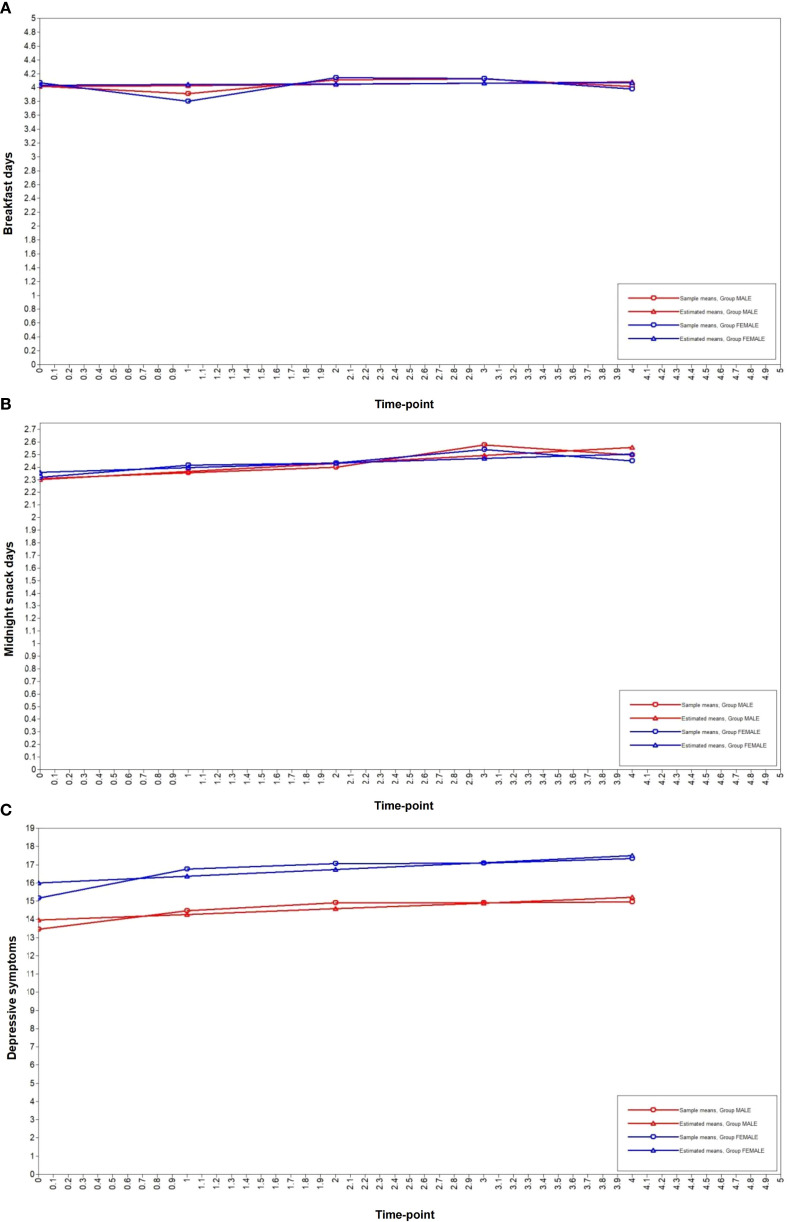
The latent growth curve of depressive symptoms, the days of eating midnight snack and breakfast per week. **(A)** breakfast days, **(B)** midnight snack days, **(C)** depressive symptoms.

The prevalence rates of depressive symptoms from T1 to T5 were 26.3%, 31.3%, 34.0%, 36.0%, and 37.3%, respectively. The reported frequencies of breakfast consumption on day 0, day 1–2, day 3–4, day 5–6, and day 7 in the past week were as follows: T1 (5.0%, 9.2%, 13.3%, 21.5%, and 51.1%, respectively), T2 (6.1%, 13.1%, 14.6%, 21.7%, and 44.5%, respectively), T3 (1.4%, 7.6%, 14.2%, 30.5%, and 46.3%, respectively), T4 (2.2%, 7.0%, 15.2%, 26.8%, and 48.7%, respectively), and T5 (4.7%, 9.2%, 14.7%, 24.6%, and 46.8%, respectively). The reported frequencies of eating a midnight snacks on day 0, day 1–2, day 3–4, day 5–6, and day 7 in the past week were as follows: T1 (28.7%, 36.3%, 18.7%, 7.6%, and 8.7%, respectively), T2 (22.9%, 40.6%, 20.3%, 7.6%, and 8.6%, respectively), T3 (19.7%, 43.4%, 20.5%, 8.5%, and 7.9%, respectively), T4 (16.6%, 41.0%, 22.1%, 10.7%, and 9.6%, respectively), and T5 (21.3%, 39.8%, 20.1%, 8.3%, and 10.5%, respectively).**Table 2** shows the fitting results of the latent growth curve model. The fitting index Akaike Information Criterion (AIC), Bayesian Information Criterion (BIC), Sample-Size Adjusted BIC, Chi-Square Test of Model Fit (*P*-Value), Root Mean Square Error Of Approximation (RMSEA), Comparative Fit Index (CFI), Tucker-Lewis index (TLI), and Standardized Root Mean Square Residual (SRMR) of the days of eating midnight snacks per week were 25170.64, 25279.32, 25215.78, 92.19 (<0.01), 0.07, 0.96, 0.96, and 0.05, respectively. These indices of the days of eating breakfast per week were 25016.14, 25124.82, 25061.28, 210.95 (<0.01), 0.11, 0.87, 0.87, and 0.09, respectively. These indices of depressive symptoms were 55776.48, 55885.17, 55821.63, 362.53 (<0.01), 0.14, 0.93, 0.93, and 0.12, respectively. **Figure 2** illustrates the potential growth trajectories of adolescents’ breakfast consumption, midnight snacks consumption, and depressive symptom scores over the past week. The variance estimates for the intercept and slope factors of breakfast consumption and depressive symptom scores were statistically significant for both males and females (all *P* < 0.01), indicating differences in initial levels and growth rates of breakfast consumption and depressive symptoms among adolescents.

**Table 2:** The latent growth curve of depressive symptoms, the days of eating midnight snack and breakfast per week.

Variable	Sex	Growth factor	Estimate	SE	Est/SE	*P*-Value
The days of eating midnight snack per week	Male	Intercept factor	0.51	0.06	8.21	<0.01
		Slope factor	0.01	0.01	1.18	0.237
	Female	Intercept factor	0.45	0.05	9.07	<0.01
		Slope factor	0.04	0.01	5.97	<0.01
The days of eating breakfast per week	Male	Intercept factor	0.45	0.06	7.63	<0.01
		Slope factor	0.03	0.01	4.37	<0.01
	Female	Intercept factor	0.37	0.05	6.86	<0.01
		Slope factor	0.03	0.01	4.05	<0.01
Depressive symptoms	Male	Intercept factor	30.39	2.51	12.13	<0.01
		Slope factor	1.73	0.25	7.10	<0.01
	Female	Intercept factor	40.29	3.03	13.31	<0.01
		Slope factor	2.35	0.26	9.120	<0.01

The variance estimates for the intercept factor and slope factors of the potential growth trajectory of midnight snacks days in the previous week were statistically significant for females (all *P* < 0.01). However, for males, the variance estimates for the intercept factor of the potential growth trajectory of male slope factors were not statistically significant (*P >* 0.05). There were differences in the initial level and growth rate of the number of midnight snacks days for females in the last week, whereas there were differences only in the initial level of the number of midnight snacks days for males in the last week, and there was no difference in the growth rate.

### Distribution of depressive symptoms among adolescents

**Table 1** presents the prevalence of depressive symptoms among adolescents with different demographic characteristics. Significant differences were observed between boys and girls at all five time points (T1: χ^2^ = 17.07, T2: χ^2^ = 27.47, T3: χ^2^ = 19.41, T4: χ^2^ = 9.66, T5: χ^2^ = 21.57; all *P* < 0.01). In addition, the prevalence of depressive symptoms varied significantly at certain time points based on family residence, ethnicity, number of close friends, self-rated family economic status, father’s education level, and father’s occupation (*P <* 0.05). However, no significant differences in the prevalence of depressive symptoms were observed across other demographic variables (*P >* 0.05).

### The association between adolescents’ breakfast behavior and midnight snacks behavior and depressive symptoms

**Figure 3:**
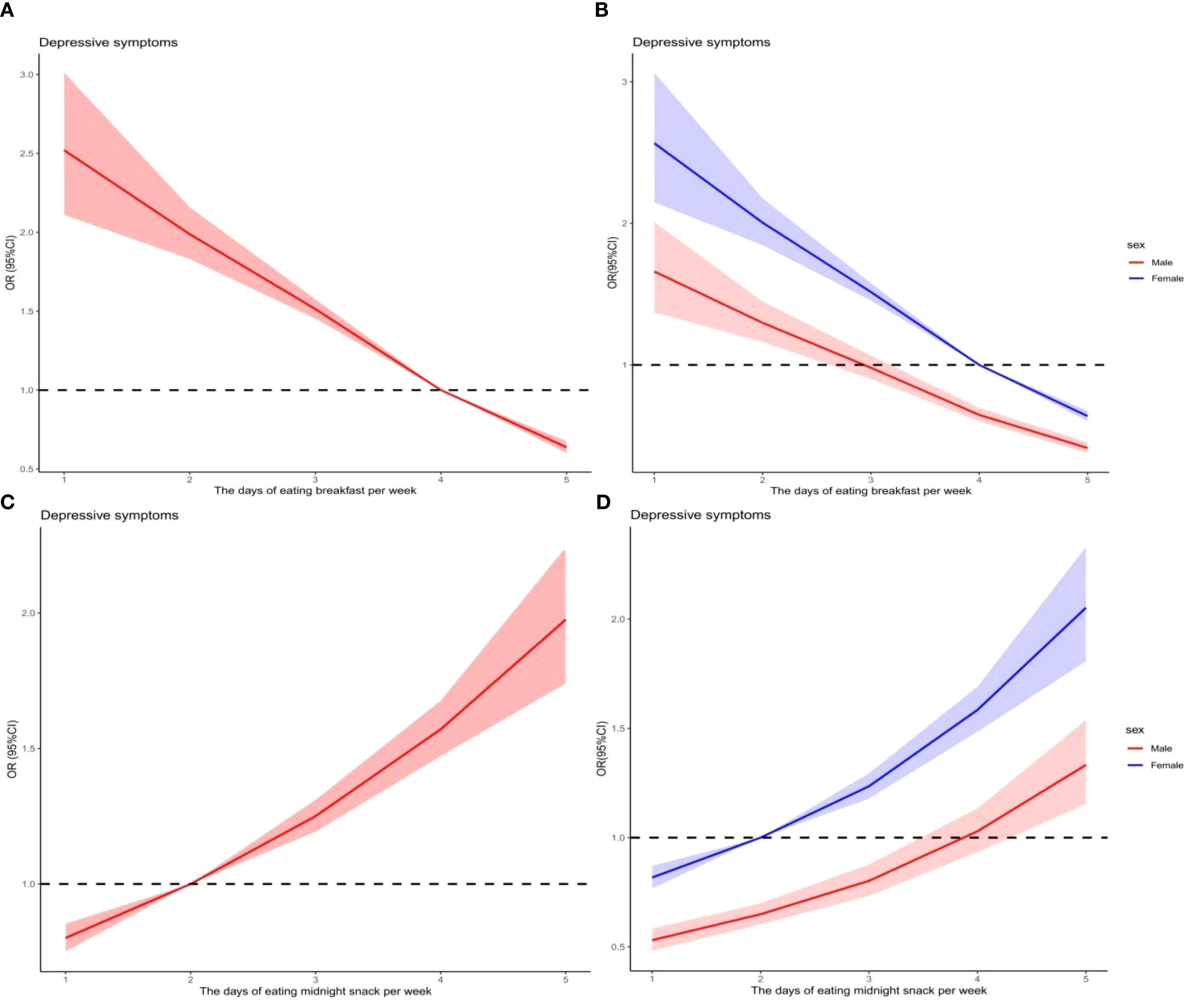
Association of breakfast and midnight snack with depressive symptoms in Yunnan multi-ethnic adolescents.

**Table 3** presents the results of the generalized linear model analysis of the association between the number of breakfast and midnight snacks days and depressive symptom scores in adolescents in the past week. After adjusting for confounding variables, the number of breakfast days was negatively associated with depressive scores across all groups: adolescents overall (*β* = -0.76, 95*%CI*: -0.87 to -0.65, *P* < 0.01), males (*β* = -0.71, 95*%CI*: -0.87 to -0.56, *P* < 0.01) and females (*β* = -0.77, 95%*CI*: -0.92 to -0.63, *P* < 0.01). In addition, the number of midnight snacks days was positively associated with depressive symptoms across all groups: adolescents overall (*β* = 0.29, 95%*CI*: 0.18 to 0.40, *P* < 0.01), males (*β* = 0.39, 95%*CI*: 0.24 to 0.55, *P* < 0.01), and females (*β* = 0.17, 95%*CI*: 0.02 to 0.32, *P* = 0.027). **Figure 3** shows the results of the restricted cubic spline analysis of the association between the number of breakfast and midnight snacks days per week and depressive symptom scores. Overall, breakfast and midnight snacks behaviors were related to adolescent depressive symptoms. Males’ breakfast behavior was more sensitive to depressive symptoms, whereas females’ midnight snacks behavior was more sensitive to depressive symptoms. Adolescents who ate breakfast fewer than four days in the past week and consumed midnight snacks more than two day per week exhibited an increased risk of depressive symptoms. Among males, eating breakfast fewer than three days per week and eating midnight snacks more than four days per week increased the risk of depressive symptoms. Among females, eating breakfast fewer than four days per week and eating midnight snacks more than two day per week was associated with an increased risk of depressive symptoms.

**Table 3:** Longitudinal association of breakfast and midnight snack with depressive symptoms in Yunnan multi-ethnic adolescents.

Model	Adolescents	Variables	*β* (95% *CI*)	SE	Wald *χ^2^*	*P*
Model 1	All participants	The days of eating breakfast per week	-0.98(-1.09−-0.87)	0.06	288.10	<0.01
		The days of eating midnight snack per week	0.51(0.39−0.62)	0.06	77.28	<0.01
	Male	The days of eating breakfast per week	-0.95(-1.11−-0.79)	0.08	131.15	<0.01
		The days of eating midnight snack per week	0.60(0.44−0.76)	0.08	54.71	<0.01
	Female	The days of eating breakfast per week	-1.00(-1.16−-0.84)	0.08	156.13	<0.01
		The days of eating midnight snack per week	0.40 (0.24−0.56)	0.08	23.86	<0.01
Model 2	All participants	The days of eating breakfast per week	-0.76(-0.87−-0.65)	0.06	185.87	<0.01
		The days of eating midnight snack per week	0.29(0.18−0.40)	0.06	26.59	<0.01
	Male	The days of eating breakfast per week	-0.71(-0.87−-0.56)	0.08	80.32	<0.01
		The days of eating midnight snack per week	0.39(0.24−0.55)	0.08	24.21	<0.01
	Female	The days of eating breakfast per week	-0.77(-0.92−-0.63)	0.07	107.73	<0.01
		The days of eating midnight snack per week	0.17(0.02−0.32)	0.08	4.89	0.027

Model 1 unadjusted for variables. Model 2 adjusted for age, ethnicity, residence, the only child in the family, family type, parental educational level, parental occupation, family changes, hospital experience, the impact of COVID-19, family history of depression, self-perceived socioeconomic status, self-perceived academic stress, video time, physical activity, smoking, alcohol consumption, the number of close friends, consumption of takeaway fast food and sugar-sweetened beverage.

## Discussion

This follow-up study revealed that the prevalence of depressive symptoms among multi-ethnic adolescents in China ranged from 26.3%-37.3%, showing a significant increasing trend. The prevalence found in this study aligns closely with that of adolescents in Eastern (36.2%) (13), as well as Bangladesh (36.6%) (52). However, the prevalence of depressive symptoms among adolescents in this study was higher than that in Northeast China (28.2%) (24) and lower than in India (52.3%) (53). Moreover, the latent growth trajectory analysis indicated that depressive symptom scores among multi-ethnic adolescents in China increased over the follow-up period. These findings are consistent with other studies, which have reported a significant increase in depression prevalence among Chinese adolescents from 2021 to 2023 (54). The results highlight that depressive symptoms among multi-ethnic adolescents in China are at a high level. While Eastern and Central China have seen societal, school, and family attention toward adolescent depression, ethnic adolescents in Western China have not received sufficient attention. Therefore, school-based health promotion programs targeting depression among multi-ethnic adolescents in China should be prioritized on the national agenda.

The reported rate of not eating breakfast for ≥1 d in the past week among Chinese multi-ethnic adolescents was 48.9%-55.5%, which is comparable to the rate in Turkish adolescents (54.1%) (29), higher than that of South Korean adolescents (29.2%) (55), and lower than that of Brazilian adolescents (87%) (56). We indicate that the number of days per week that males and females ate breakfast was negatively correlated with the risk of developing depressive symptoms. Specifically, the risk of depressive symptoms increased when males ate breakfast less than three days per week, and females ate breakfast less than four days per week. These results align with previous studies. For instance, a cross-sectional study found that Turkish adolescents who ate breakfast ≤5 days per week were at a higher risk for depressive and anxiety symptoms, and low breakfast quality was also linked to these mental health issues (29). Studies have shown that skipping breakfast is associated with higher levels of depressive symptoms. Spanish individuals who did not eat breakfast exhibited more depressive symptoms than those who did (28). Among Japanese adolescents, regular breakfast consumption (≥6 days per week) was negatively correlated with depressive symptoms (26, 27). A Chinese study found a similar pattern, with a stronger association observed in younger adolescents (11–15) compared to older ones (16–19) (33). Additionally, research on children in grades 1–9 in Shanghai, China, indicated that adolescents who skipped breakfast had a 2.70 times higher risk of depressive symptoms than those who ate breakfast regularly (45). Few longitudinal studies have examined this relationship. However, a one-year prospective cohort study in China found that compared to adolescents who ate breakfast ≥6 days per week, those who ate breakfast only 2–5 days per week had a significantly higher risk of depressive symptoms (*OR* = 2.045), with an even greater risk for those eating breakfast ≤1 day per week (*OR* = 2.722) (32). Similarly, a large-sample prospective study in the United States confirmed a positive association between breakfast and depressive symptoms (57). Moreover, skipping breakfast has been linked to an increased risk of suicidal ideation, planning, and attempts among adolescents in the United States and South Korea (58, 59). Given these findings, promoting consistent breakfast consumption among adolescents is crucial for mental health.

This study found that 71.3%-83.4% of multi-ethnic Chinese adolescents consumed midnight snacks at least once in the past week, a significantly higher rate than that of Bangladeshi (16.6%) (36) and Palestinian (29.7%) adolescents (37). Additionally, our findings indicate a positive correlation between the frequency of midnight snacks consumption and the risk of depressive symptoms in both males and females. The risk of depressive symptoms increased when males consumed midnight snacks more than four days per week and females more than two day per week. This finding aligns with previous research indicating that midnight snacks are associated with a higher risk of depressive symptoms in adolescents (41, 43). Similarly, children and adolescents in grades 1–9 in Shanghai, China who ate before bedtime showed a significantly elevated risk of depressive symptoms (45). Turkish adolescents aged 13–18 who ate after 10 PM were also more likely to experience depressive symptoms (42). Similarly, a longitudinal study in Montreal that followed Canadian adolescents from ages 12 to 17 found that nighttime eating was longitudinally associated with an increased risk of depressive symptoms (*OR* = 1.75) (35).

Our research data indicate the gender-specific thresholds for skipping breakfast and eating midnight snacks increase the risk of depressive symptoms, the possible explanations include Physiological and social-cultural factors. Meta-analysis shows that the gender difference for diagnoses of depression emerged earlier than previously thought, peaked in adolescence, but then declined and remained stable in adulthood (60). The Social Signal Transduction Theory posits that adolescent females are more sensitive to experiences of social-environmental adversity. Moreover, fluctuations in hormone levels can regulate the sensitivity of females to stress, as well as the structure and function of the brain, and their reactivity, which leads to a higher likelihood of depressive symptoms in adolescent females (61).

In this study, skipping breakfast and eating midnight snacks among multi-ethnic Chinese adolescents were longitudinally associated with depressive symptoms. Several mechanisms may explain this relationship. First, both behaviors negatively impact physical health, which serves as the foundation for mental well-being. Breakfast is a crucial meal of the day, providing essential energy and nutrients that contribute significantly to daily dietary intake. Breakfast typically includes grains, dairy products such as cheese or milk, and eggs. Whole grains are rich in several macronutrients, including magnesium (62); dairy products are excellent sources of Calcium, magnesium and vitamins (such as B_2_, B_12_, A), amino acids (such as proline, tyrosine, serine, lysine, valine, and leucine) (63); and eggs provide a high amount of choline (64). These nutrients may contribute positively to mental health. Consistently skipping breakfast can result in nutrient deficiencies, while frequent midnight snacks places excessive strain on the gastrointestinal tract, potentially leading to indigestion. Additionally, frequent midnight snacks can contribute to obesity, increasing the likelihood of adolescents entering a sub-health state. Research indicates that sub-health serves as a mediating factor in the relationship between dietary behaviors and depressive symptoms (65). Second, both skipping breakfast and eating midnight snacks can negatively impact sleep quality and even contribute to insomnia (33, 66). Previous studies have suggested that night eating symptoms have a direct effect on the chronotype differences and insomnia and an indirect effect on disordered eating attitudes, by increasing insomnia scores (67). Midnight snacks keeps the stomach full, disrupts sleep, and is a significant predictor of depressive symptoms (68). Third, irregular eating habits, such as skipping breakfast and frequently eating midnight snacks, can disrupt the biological clock and lead to endocrine imbalances, which may further contribute to depressive symptoms (69). Fourth, most midnight snacks consumed by adolescents are ultra-processed foods that are easy to access and are significant predictors of depressive symptoms (70). Nighttime eating is also linked to food addiction (71). These foods—such as sugar-sweetened beverages, processed meat, sweets, and snacks—are frequently consumed, further increasing the risk of depressive symptoms in adolescents (23).

The data collected in this study were during the COVID-19 pandemic. Therefore, the depressive symptoms of adolescents might have been affected by COVID-19. A nationwide, multicenter, cross-sectional study in China revealed that the COVID-19 pandemic has exacerbated anxiety, depression, and sleep problems among people (72). Moreover, social isolation and reduced outdoor activities have emerged as significant risk factors for anxiety, depression, and insomnia (73). However, we have taken into account the impact of the COVID-19 pandemic on the participants’ depressive symptoms, and in the multivariate analysis model, we have adjusted for the COVID-19 pandemic.

Adolescent depression is a pressing global public health concern (74). It not only impacts adolescents’ well-being but also affects their long-term health and that of future generations (60). From a life-cycle perspective, as well as in terms of treatment effectiveness and health economics, preventing adolescent depression is crucial (1). The link between adolescent dietary behaviors and depressive symptoms has garnered increasing attention (75). Research suggests that dietary interventions can effectively prevent depression or serve as an alternative or adjunct therapy (76). Therefore, the habits of skipping breakfast and eating midnight snacks among multi-ethnic adolescents in China require attention. Effective health education and behavioral interventions should be implemented to correct these unhealthy eating patterns. Adolescents should be encouraged to eat a balanced breakfast regularly, maintain consistent meal timing, and develop healthy dietary habits to support mental well-being.

Emerging evidence suggests a bidirectional relationship between dietary behaviors and depressive symptoms among adolescents. Specifically, depressive symptoms may adversely influence eating behaviors, leading to poorer nutritional choices (77). Empirical research supports this association, demonstrating a significant link between depression and uncontrolled eating behaviors (*β* = 0.61) (78). Consistent with these findings, a Brazilian study involving college students revealed that elevated depressive symptom scores were correlated with increased consumption of hyperpalatable foods, indicating a preference for energy-dense, nutrient-poor diets (79). Further corroborating this trend, an Indian longitudinal study observed that adolescents with depressive symptoms exhibited a higher propensity for unhealthy dietary practices, subsequently elevating their risk of weight gain (80). We will further explore the association of depressive symptoms with breakfast and midnight snacks in China multi-ethnic adolescents.

This study has several strengths. First, it explored the longitudinal relationship between breakfast and midnight snacks behaviors and depressive symptoms among multi-ethnic adolescents in China, reducing the risk of spurious correlations often found in cross-sectional studies. Our research findings hold practical value for developing intervention strategies aimed at modifying unhealthy dietary behaviors among multi-ethnic adolescents in Yunnan Province, fostering healthy eating habits, and promoting psychological well-being. Second, this is the first study to examine the breakfast and midnight snacks habits of multi-ethnic adolescents in Yunnan, China, and their impact on depressive symptoms, addressing a gap in existing research on this population.

This study has some limitations. First, self-reported questionnaires used to assess dietary behaviors and depressive symptoms may introduce recall bias or social desirability bias, particularly among adolescents. throughout the study, we implemented several strategies to mitigate such biases. Second, the study focuses only on the frequency of breakfast and midnight snacks consumption while ignoring critical factors like the nutritional content of breakfast and the type of midnight snacks, limiting the interpretation of whether associations stem from quantity or quality, which may limit the precision of the analysis. Third, although the study adjusted for confounders such as demographics and lifestyle factors, it omitted other relevant variables (e.g., family dietary habits, peer influence, specific nutrient deficiencies), which may leave residual confounding. Fourth, this study has a limited sample size and was conducted only in specific regions of Yunnan Province, which may not fully represent all Yunnan. Consequently, the generalizability and application of the findings are constrained.

## Conclusion

In conclusion, our longitudinal data indicate that skipping breakfast and eating midnight snacks are significant predictors of depressive symptoms among multi-ethnic adolescents in China. These findings underscore the importance of implementing school-based health education and behavioral interventions. By guiding students to adopt healthy dietary habits—such as eating breakfast regularly and avoiding midnight snacks—depressive symptoms can be prevented and improved, ultimately promoting better health among multi-ethnic adolescents in China.

## Data Availability

The raw data supporting the conclusions of this article will be made available by the authors, without undue reservation.
